# Hospital use of systemic antifungal drugs

**DOI:** 10.1186/1472-6904-5-1

**Published:** 2005-02-10

**Authors:** Katja de With, Michaela Steib-Bauert, Holger Knoth, Frank Dörje, Egid Strehl, Ulrich Rothe, Ludwig Maier, Winfried V Kern

**Affiliations:** 1 Center for Infectious Diseases and Travel Medicine, University Hospital, D-79106 Freiburg, Germany; 2 Academic Medical Center Pharmacy, Technical University, Dresden, Germany; 3 Pharmacy Service, Hospital of the Friedrich-Alexander University, Erlangen, Germany; 4 Pharmacy Service, University Hospital, Freiburg, Germany; 5 Pharmacy, University Hospital, Regensburg, Germany; 6 Pharmacy, Ulm University Hospital and Medical Center, Ulm, Germany

## Abstract

**Background:**

Sales data indicate a major increase in the prescription of antifungal drugs in the last two decades. Many new agents for systemic use that only recently have become available are likely to be prescribed intensively in acute care hospitals. Sales data do not adequately describe the developments of drug use density. Given the concerns about the potential emergence of antifungal drug resistance, data on drug use density, however, may be valuable and are needed for analyses of the relationship between drug use and antifungal resistance.

**Methods:**

Hospital pharmacy records for the years 2001 to 2003 were evaluated, and the number of prescribed daily doses (PDD, defined according to locally used doses) per 100 patient days were calculated to compare systemic antifungal drug use density in different medical and surgical service areas between five state university hospitals.

**Results:**

The 3-year averages in recent antifungal drug use for the five hospitals ranged between 8.6 and 29.3 PDD/100 patient days in the medical services (including subspecialties and intensive care), and between 1.1 and 4.0 PDD/100 patient days in the surgical services, respectively. In all five hospitals, systemic antifungal drug use was higher in the hematology-oncology service areas (mean, 48.4, range, 24 to 101 PDD/100 patient days, data for the year 2003) than in the medical intensive care units (mean, 18.3, range, 10 to 33 PDD/100) or in the surgical intensive care units (mean, 10.7, range, 6 to 18 PDD/100). Fluconazole was the most prescribed antifungal drug in all areas. In 2003, amphotericin B consumption had declined to 3 PDD/100 in the hematology-oncology areas while voriconazole use had increased to 10 PDD/100 in 2003.

**Conclusion:**

Hematology-oncology services are intense antifungal drug prescribing areas. Fluconazole and other azol antifungal drugs are the most prescribed drugs in all patient care areas while amphotericin B use has considerably decreased. The data may be useful as a benchmark for focused interventions to improve prescribing quality.

## Background

There has been a major increase in the prescription of antifungal drugs after the introduction of fluconazole into the market in the late 1980s, and again in the late 1990s. The systemic antifungal market has continued to experience growth since 1999, increasing in value from $2.1 billion to $3.3 billion in 2003. The azoles dominate the systemic antifungal market, accounting for 52% of total sales in 2003 [1-8]. The reasons for the increasing antifungal drug use are manifold. Among hospitalized patients, the empiric use of antifungals in both hematology-oncology as well as intensive care patients is now common. Often, treatment is initiated based on preliminary microbiology results, and definite diagnosis of invasive infection versus colonization may be difficult [4,9-11]. New antifungal drugs such as itraconazole, caspofungin, and voriconazole have become available and broadened therapeutic options [12]. In some settings an increasing incidence of invasive fungal infections and the emergence of infections due to rare and atypical organisms has been observed, and this changing epidemiology has contributed to more intense use of antifungal drugs [13]. In the ambulatory care setting there was a shift from prescribing intravaginal antifungal preparations to fluconazole over-the-counter, raising concern about the possible development of azole drug resistance [14-16].

Although multiple current and projected market and sales data on systemic antifungal drugs are available, few studies have provided estimates of antifungal drug use density especially in hospitals. Alvarez-Lerma and colleagues reported a prescription rate of 14% in intensive care unit patients [9]. In a survey we conducted in 1994 the prescription prevalence rate in hospitalized patients was 10.2% per patient-week in the medical service and 3.5% per patient-week in the surgical services [17]. Hospital expenditures were also evaluated in some studies. However, we were unable to find information on recent hospital antifungal drug utilization that uses the daily doses per 100 patient days format which is now common in pharmacoepidemiologic surveys. We therefore collected data from the pharmacies of five university hospitals and here report overall and comparative use density values for defined patient care areas.

## Methods

Pharmacy data on systemic antifungal drug use in the medical and surgical services of five university hospitals located across Germany were obtained for the period 2001 to 2003. The five university hospitals included, here designated A through E, varied in size from ~1,000 to ~1,700 beds, and differed from each other in structure, special services offered, and in the availability of interdepartmental guidelines and an antiinfective therapeutics committee, drug formularies, formulary restrictions, and infectious disease consultation services.

We used a consensus definition of (usually) prescribed daily doses (PDD) in adults (Table 1) according to local guidelines. This definition differs from the daily doses defined by the WHO/ATC classification  which defines lower doses for amphotericin B, fluconazole, and itraconazole (Table 2). Antifungal drug use density was calculated as yearly PDD/100 patient days (i.e. occupied bed days). Separate data were calculated for the medical ICU (MICU), the surgical ICU (SICU), and the hematology-oncology services, respectively. We also calculated yearly means of overall and specific antifungal use densities to assess time trends.

**Table 1:** Definitions of prescribed daily doses (PDD) and WHO/ATC defined daily doses (DDD) for systemic antifungal drugs.

	PDD	DDD
amphotericin B deoxycholate*	50 mg	35 mg
liposomal amphotericin B	250 mg	nd^#^
flucytosin	10 g	10 g
ketoconazole	400 mg	400 mg
fluconazole	400 mg	200 mg
itraconazole	400 mg	200 mg
voriconazole	400 mg	400 mg
caspofungin	50 mg	50 mg

*conventional amphotericin B

^#^not defined

## Results and discussion

**Figure 1:**
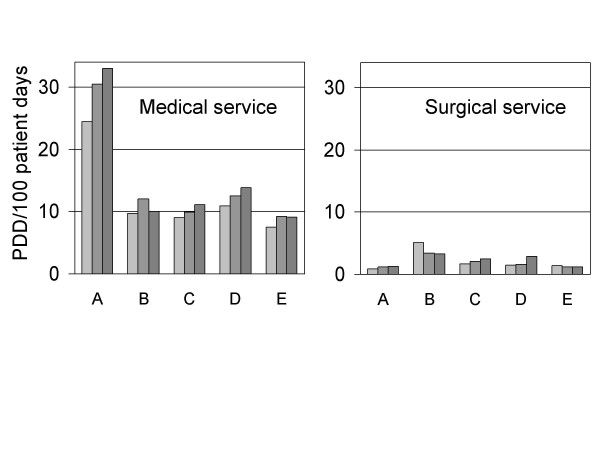
Yearly systemic antifungal drug use density in the medical and surgical services of five university hospitals (A through E) for the years 2001–2002–2003.

The yearly antifungal drug use densities differed between the five hospitals in particular for the medical services. Hospital A showed use density values of consistently >20 PDD/100 patient days while hospital E values were consistently <10 PDD/100 patient days (Figure 1). Less variation between the hospitals were observed in the surgical services (Figure 1). Here, 3-year averages for the hospitals ranged between 1.1 (hospital A) and 4.0 PDD/100 patient days (hospital B), respectively.

### Time trend

Overall, the mean antifungal drug use for the five hospitals increased between the years 2001 and 2003 from 12.4 to 15.4 PDD/100 patient days in the medical services (+24%), but only from 2.1 to 2.2 PDD/100 patient days in the surgical services (+5%). Applying the WHO/ATC definition of daily defined doses (DDD; including our daily dose definition for liposomal amphotericin B), corresponding values for the years 2001 and 2003 were calculated to be 22.8 to 26.3 DDD/100 patient days (+15%) in the medical services, and 4 to 4.1 DDD/100 patient days (+4%) in the surgical services, respectively (data not shown).

### Use of specific antifungal drugs

**Figure 2:**
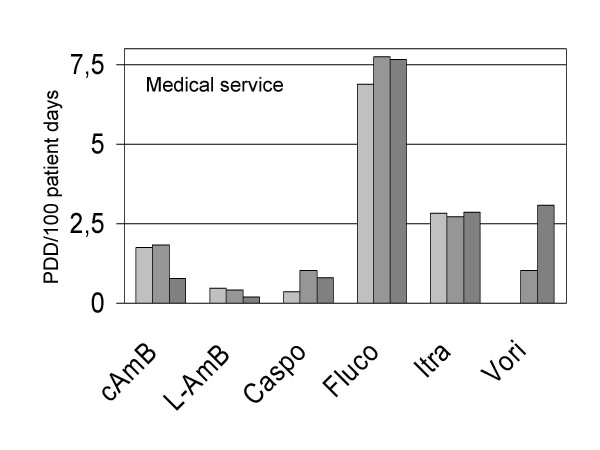
Use density for different antifungal drugs in the medical service of five university hospitals. Data are yearly means for 2001, 2002 and 2003. cAmB, conventional amphotericin B; L-AmB, liposomal amphotericin B; Caspo, caspofungin; Fluco, fluconazole; Itra, itraconazole; Vori, voriconazole.

As in other reports [5], fluconazole was the most frequently prescribed antifungal drug in the medical as well as surgical services of the five hospitals. Its use did not decrease over time. Figure 2 shows the yearly mean use density for fluconazole and other antifungal drugs (except the rarely used 5-flucytosin and ketoconazole) in the medical service. Interestingly, conventional as well as liposomal amphotericin B use decreased over time (Figure 2). In the year 2003, the mean use of fluconazole in the medical service was 7.7 PDD/100 patient days (representing 50% of all PDDs), and 1.8 PDD/100 patient days in the surgical service (representing 78% of all PDDs), respectively.

### Differences between patient care areas

**Figure 3:**
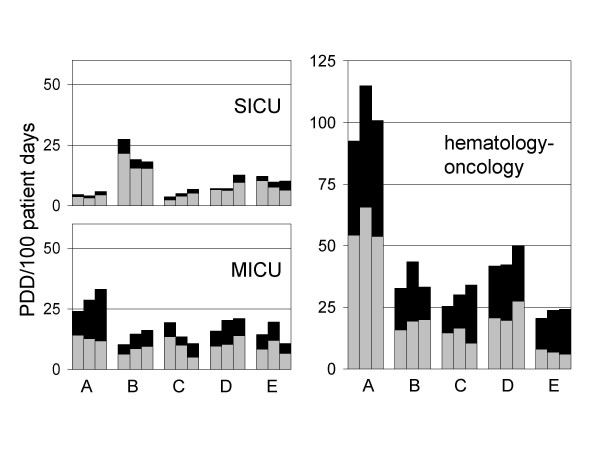
Use of fluconazole (grey bars) versus other systemic antifungal drugs (black bars) in the SICU, MICU, and in the hematology-oncology services of five university hospitals (A through E) during the years 2001–2002–2003.

As expected, antifungal drug use was much more intense in the hematology-oncology services and intensive care areas (Figure 3) than in general internal medicine (mean use, 2.3 PDD/100 patient days, data for the year 2003) and general surgery (mean use, 1.1 PDD/100 patient days, data for the year 2003). Figure 3 shows that there was some variation between the hospitals in the use density values, particularly in hematology-oncology and the SICU area. These differences were not explained by different incidences of invasive fungal infections as perceived by the local physicians, but in none of the hospitals specific surveillance for fungal infections was activated. Large differences were also noted in the fluconazole use, with very high use density values in hospital A hematology-oncology and comparatively low density values in hospital E hematology-oncology areas (53.8 versus 5.8 PDD/100 patient days, data for the year 2003). The high use density values in hospital A hematology-oncology could primarily be explained by the heavy use of relatively high doses of fluconazole (400 mg daily) for prophylactic purposes which was much less common in the other hospitals.

Of note, hospital E had a moderately active infectious disease consultant service with an antimicrobial agents management program, and this was previously associated with low antibacterial drug use in the medical service [18,19]. According to the present study, this programme was also perhaps linked to the low antifungal drug use density in the hospital E medical service including hematology-oncology.

In hospital C, there was a program in the MICU attempting to decrease the use of fluconazole based solely on positive cultures for yeasts in tracheal or bronchial secretions. This program, which was primarily a focused infectious diseases consultation program was started in 2002, and appeared to be effective in decreasing fluconazole use from 13.5 to 5 PDD/100 patient days without changing the use density of other systemic antifungal drugs (Figure 3).

The decreasing use of amphotericin B consumption seen in the medical service was to a large part explained by decreasing use of the drug in the hematology-oncology wards. Mean use density values changed between 2001 and 2003 from 5.8 to 2.4 PDD/100 patient days for conventional amphotericin B, and from 1.6 to 0.6 for liposomal amphotericin B, respectively. These changes were associated with increasing values for voriconazole in hematology-oncology. This new drug after its introduction into the market in 2002 increased from zero to a use density of 10.3 PDD/100 patient days in 2003. Interestingly. 80% of all doses of voriconazole in hematology-oncology were by the oral route.

## Limitations and conclusions

Our study was not designed to evaluate appropriateness of antifungal drug therapy. Few studies in the hospital setting have addressed this issue. In two previous studies, it was found that dosages of fluconazole were not always adequate [20,21]. In another study, therapy was considered "unconventional" in 27% of the courses and 41% of the regimens, mainly because either the indication or the duration of treatment did not conform to conventional practice [4]. Conventional practice, however, can differ considerably as indicated by our results. We think it is unlikely that the observed high use density values in hospital A hematology-oncology (>50 PDD/100 patient days) represents an unusual epidemiologic situation or a major difference in hematology-oncology patient-mix. Rather, the intense use can be explained by liberal antifungal drug use in high doses for prophylaxis and perhaps empiric combination therapy. The present study, thus, provided a useful benchmark suggesting that more detailed analysis of antifungal therapy indication practice is warranted in this particular hospital.

In summary, this report describes the range of antifungal drug use in certain patient care areas of large tertiary-care teaching hospitals in Germany. Consistent with other reports, we found that fluconazole has remained the most frequently prescribed drug in this setting.

## Competing interests

The author(s) declare that they have no competing interests.

## Authors' contributions

KdW and WVK analysed and interpreted the data and wrote the article. MSB is data manager, analysed the data and presented them through a searchable database. HK, FD, ES, UR and LM checked the data for consistency and correctness, provided them in electronic format, and helped with interpretation of the data and revision of the manuscript.

## Pre-publication history

The pre-publication history for this paper can be accessed here:


